# *Caveolin-1* rs1997623 variant and adult metabolic syndrome—Assessing the association in three ethnic cohorts of Arabs, South Asians and South East Asians

**DOI:** 10.3389/fgene.2022.1034892

**Published:** 2022-10-21

**Authors:** Ashraf Al Madhoun, Prashantha Hebbar, Rasheeba Nizam, Dania Haddad, Motasem Melhem, Mohamed Abu-Farha, Thangavel Alphonse Thanaraj, Fahd Al-Mulla

**Affiliations:** ^1^ Genetics and Bioinformatics, Dasman Diabetes Institute, Kuwait City, Kuwait; ^2^ Animal and Imaging Core Facilities, Dasman Diabetes Institute, Kuwait City, Kuwait; ^3^ Special Services Facility, Dasman Diabetes Institute, Kuwait City, Kuwait

**Keywords:** caveolin-1, rs1997623, MetS, metabolic syndrome, ethnicity, Arab, South Asian, Southeast Asian

## Abstract

**Background:** Animal and cell model studies have implicated *CAV1* in the pathophysiology of metabolic disorders. Our previous studies demonstrated a potential association of *CAV1* rs1997623 C/A variant with pediatric metabolic syndrome (MetS) in Arab children. In the present study, we evaluate whether the *CAV1* variant associates with MetS Arab adults as well. The association signal is further examined for ancestry-specific variation by considering cohorts of other ethnicities.

**Method:** The *CAV1* rs1997623 was genotyped in three cohorts of Arab (*n* = 479), South Asian (*n* = 660), and South East Asian (*n* = 362) ethnic adults from Kuwait. MetS status of the individuals was diagnosed using the IDF criteria (presence of central obesity and at least two abnormalities out of: elevated TG, low HDL, hypertension, or T2D). The quantitative measure of MetS was calculated as siMS = 2 × WC/Height + FBG/5.6 + TG/1.7 + SBP/130–HDL/1.02 for males or HDL/1.28 for females. Allelic associations with quantitative and dichotomous MetS traits were assessed using linear and logistic regression models adjusted for age and sex. In addition, empirical *p-*values (P_*emp*_) were generated using max(T) permutation procedure based on 10,000 permutations.

**Results:** The *CAV1* variant was significantly associated with MetS status (OR = 1.811 [1.25–2.61]; *p*-value = 0.0015; P_*emp*_ = 0.0013) and with siMS (Effect size = 0.206; *p*-value = 0.0035; P_*emp*_ = 0.0028) in the cohort of Arab individuals. The association was weak and insignificant in the South Asian and South East Asian cohorts (OR = 1.19 and 1.11; *p*-values = 0.25 and 0.67, respectively).

**Conclusion:** The reported association of *CAV1* rs1997623 C/A with MetS in Arab pediatric population is now demonstrated in an adult Arab cohort as well. The weak association signal seen in the Asian cohorts lead us to propose a certain extent of ethnic-specificity in *CAV1* rs1997623 association with MetS.

## Introduction

Metabolic syndrome (MetS) in adults is defined as a cluster of risk factors for cardiovascular disease and type 2 diabetes mellitus, which includes abdominal obesity, dyslipidemia, glucose intolerance, hypertension, stroke, and atherosclerosis. The global epidemic proportions of MetS are estimated to be around 20–25%, with differences seen across continents–*e.g.*, 12–37% of the Asian population versus 12–26% of the European population are afflicted with MetS (Alberti et al., 2009). The prevalence of MetS in the Arabian Gulf Region is reported as 27.3% (Shin and Jee, 2020). An alarming increase in pediatric MetS has been observed among Arab children and adolescents over 9 years. For instance, in Saudi Arabia, the prevalence of pediatric MetS was 11.8% in 2010 and increased to 20.6% in 2019 (Amer et al., 2021)**.**

MetS is triggered by various factors that include genetic predisposition, aging, obesity, insulin resistance, and physical inactivity. Genetic predisposition is a factor in MetS as prevalence differs among ethnic groups (Ford et al., 2002). Substantial progress has been made over the last decade in identifying the genetic risk factors associated with various traits of MetS (Abou Ziki and Mani, 2016). One of these risk loci is the *Caveolin-1* (*CAV1*) gene, its association with metabolic disorders has been demonstrated in animal models and human cohorts (Baudrand et al., 2016; Chen et al., 2016). *CAV1* is an essential structural protein required for the formation of caveolae (Vargas et al., 2002; McMahon et al., 2009), which play key roles in cellular homeostasis including the regulation of influx and efflux of lipid and glucose metabolites in various tissues (Su and Abumrad., 2009; Pilch and Liu, 2011; Grayson et al., 2013). *CAV1* has been widely studied in metabolic syndrome disorders such as dyslipidemia and CVD due to signal transduction, trafficking in cholesterol hemostasis, and triacylglycerol metabolism (Jin et al., 2011).

Several studies have indicated that *CAV1* genetic variations might interact with other risk factors (Haddad et al., 2020), including dietary intake of fatty acids, suggesting a positive association between *CAV1* and hypercholesterolemia (Shyu et al., 2017). A study using whole exome sequencing of a family of European descent with pulmonary arterial hypertension identified a frame-shift mutation in *CAV1* (c.474delA, P158PfsX22) among nine family members (Austin et al., 2012). *CAV1* rs926198 has been seen associated with increased susceptibility to coronary artery disease and myocardial infarction in Han Chinese population (Chen et al., 2016). Baudrand et al. (2015) found the CAV1 gene variant rs926198 associated with MetS in separate Caucasian and Hispanic cohorts. In 2011, Pojoga et al., 2011 demonstrated the association of CAV1 rs926198 and rs3807989 variants with higher fasting insulin levels and increased homeostatic model assessment for insulin resistance (HOMA-IR) in Caucasian and Hispanic cohorts. Mora-Garcia et al. (2018) found the CAV1 variation rs11773845 to be associated with high serum triglycerides (TG) and MetS in a Latin American cohort.

Recently, we reported an association between the *CAV1* rs1997623 variant and MetS in a cohort of Kuwaiti children (Nizam et al., 2018). Evaluation of this association has not yet been performed in adults. Cardiovascular risk factors, comprising MetS, can appear in childhood and progress through adulthood, leading to higher cardiovascular morbidity in adults (Varda and Gregoric, 2009). In this study, we assess whether the association signals observed between the *CAV1* rs1997623 variant and pediatric MetS are also observed in adult MetS. We further evaluate the association signal involving the rs1997623 variant for ethnic variation by analyzing additional South Asian and South East Asian cohorts.

## Materials and methods

The studies involving human participants were reviewed and approved by Ethical Review Committee, Dasman Diabetes Institute, Kuwait. The patients/participants provided their written informed consent to participate in this study.

### Type of study

The study is of transverse observational type.

### Recruitment of participants and study cohort

Adult individuals of Arab ethnicity from Kuwait were recruited as study subjects for the Arab cohort; of South Asian ethnicity for the South Asian cohort; and of South East Asian ethnicity for the South East Asian cohort. Individuals aged >18 years and residents of Kuwait were included. Pregnant women and individuals with Mendelian or rare genetic disorders, cognitive deficiencies, or chronic disorders (such as cancer) were excluded. The Arab cohort comprised 479 Arab subjects; the South Asian cohort comprised 660 South Asian individuals; and the South East Asian cohort comprised 362 South East Asian individuals. For each participant upon enrolment, data on demographics, health disorders (obesity, diabetes, and hypertension), baseline characteristics (such as height, weight, waist circumference, and blood pressure), and use of medication (such as those for lowering lipid levels or treating diabetes and hypertension) were recorded.

### Derivation of MetS status and siMS score

We adopted the adult diagnosis criteria defined by the International Diabetes Federation (Alberti et al., 2006) to derive the MetS status of every study participant. To diagnose the patients with MetS, they should have central obesity and two or more of the following: high TG, low high-density lipoprotein (HDL), hypertension, or raised fasting plasma glucose (FPG). An individual was considered to have 1) central obesity if the waist circumference (WC) was ≥94 cm in the case of men or ≥80 cm in the case of women, 2) high TG if the serum TG level was ≥1.7 mmol/L (151 mg/dl) or was taking medication to lower TG; 3) low HDL if the serum HDL cholesterol level was < 1.03 mmol/L (40 mg/dl) in the case of men or < 1.29 mmol/L (50 mg/dl) in the case of women or was taking medication to restore HDL levels; 4) hypertension if systolic blood pressure was ≥130 mm Hg, diastolic blood pressure was ≥85 mm Hg, or was having treatment for previously diagnosed hypertension; and 5) diabetes if FPG was ≥5.6 mmol/L (100 mg/dl), or was previously diagnosed for type 2 diabetes. Furthermore, we calculated the siMS score, which is a simple and accurate method for quantifying MetS in adults, using the formula described in (Soldatovic et al., 2016): siMS score = 2*WC/Height + FBG/5.6 + TG/1.7 + SBP/130-HDL/1.02 for men or HDL/1.28 for women.

### Blood sample collection and processing

After confirming that the participant had fasted overnight, blood samples were collected in EDTA-treated tubes. DNA was extracted using Gentra Puregene kit (Qiagen, Valencia, CA, United States) and was quantified using Quant-iT™ PicoGreen dsDNA Assay Kit (Life Technologies, Grand Island, NY, United States) and Epoch Microplate Spectrophotometer (BioTek Instruments). We checked absorbance values at 260–280 nm for adherence to an optical density range of 1.8–2.1 as previously described (Nizam et al., 2018).

### Targeted genotyping of the *CAV1* study variant rs1997623

Candidate SNP genotyping was performed using the TaqMan Genotyping Assay kit (C_2972973_10 from Thermo Fisher Scientific Inc, United States) on ABI 7500 Real-Time PCR System (Applied Biosystems, Foster City, CA, United States). 10 ng of DNA and 5 × FIREPol Master Mix (Solis BioDyne, Estonia) was mixed with 1 µl of 20 × TaqMan SNP Genotyping Assay to form the polymerase chain reaction sample. Thermal cycling conditions were set at 60°C for 1 min and at 95°C for 15 min followed by 40 cycles of 95°C for 15 s and 60°C for 1 min. Sanger sequencing was performed, using the BigDye Terminator v3.1 Cycle Sequencing on Applied Biosystems 3730xl DNA Analyzer, for selected cases of homozygotes and heterozygotes to validate genotypes determined by the above techniques (Nizam et al., 2018).

### Quality procedures for SNP and trait measurements

SNP quality and statistical associations with traits were assessed using PLINK (version1.9) (Purcell et al., 2007). Minor allele frequency (MAF) was calculated in the study cohort and compared with those reported for ethnic populations in the 1000 Genomes Project (Auton et al., 2015). Hardy–Weinberg equilibrium was calculated for the studied variant. Quantitative trait values < Q1- 1.5 * IQR or > Q3+1.5 * IQR were treated as outliers and excluded from further analysis.

### Association tests and empirical p-values to ascertain statistical significance

Allelic association tests for the studied variant with quantitative traits (such as siMS score) and dichotomous traits (such as MetS status) were performed using linear and logistic regression, respectively. The tests were performed under a genetic model based on the additive mode of inheritance. The tests were adjusted for regular corrections towards age and sex. We also adjusted for diabetes medication and lipid-lowering medication. Empirical *p-*values (P_*emp*_) were generated using the max(T) permutation procedure available in PLINK, based on 10,000 permutations. A threshold of < 0.05 was set for both the *p*-value and P_*emp*_ value to assess the statistical significance of the association signal.

### Power calculation

We used the Quanto software tool (University of Southern California, Los Angeles, CA, United States) to calculate the power of the study cohorts and its ability to delineate quantitative trait variability at a given power (which was set at 80%). We considered additive mode as the underlying genetic model with “gene only” hypothesis at type 1 error, *p* ≤ 0.05. Genetic effect that accounts for at least 0.1%–5% variance in the trait was detected by assuming R_G_^2^ (estimate for marginal genetic effect) values in the range of 0.001–0.05 in step of 0.005. We considered the populations (mean ± standard deviation) of the quantitative trait and MAF in these calculations.

## Results

### Characteristics of the study cohorts and study variant

Participants with MetS in our study cohorts differed significantly from those free of the syndrome in the levels of metabolic traits (except in height, TC, and LDL) and in the status for metabolic disorders (obesity, diabetes, and hypertension) in each of the cohorts of Arabs, South Asians, and South East Asians (Table 1). Sub-cohorts of MetS individuals and MetS-free individuals, though did not differ in sex, differed significantly in age; the results of association tests were subsequently adjusted for age and sex.

**TABLE 1:** Descriptive statistics for MetS and MetS-free individuals in the study cohorts of Arab, South Asian and South East Asian individuals from Kuwait.

Phenotype	Cohorts^a^								
	Arab			South asian			South east asian		
	MetS-free (N = 307)	MetS (N = 172)	*p*-value^b^	MetS-free (N = 447)	MetS (N = 213)	*p*-value^b^	MetS-free (N = 217)	MetS (N = 145)	*p*-value^b^
Sex (Male: Female)	59.0%:41.0%	47.1%:52.9%	0.012	73.6%:26.4%	68.8%: 31.2%	0.199	27.6%:72.4%	44.9%: 55.1%	< 0.001
Age (years)	42.50 ± 11.60	48.57 ± 10.17	< 0.001	42.02 ± 9.86	44.91 ± 9.07	< 0.001	39.62 ± 9.57	43.33 ± 8.62	< 0.001
Height (cm)	165.08 ± 8.50	164.13 ± 8.70	0.033	165.75 ± 7.78	165.91 ± 9.09	0.817	156.79 ± 7.33	158.82 ± 9.07	0.020
Weight (Kg)	81.90 ± 16.20	89.35 ± 13.90	< 0.001	71.32 ± 11.13	78.66 ± 9.97	< 0.001	63.18 ± 11.80	70.49 ± 12.69	< 0.001
BMI (Kg/m^2^)	29.90 ± 5.840	33.28 ± 5.00	< 0.001	25.95 ± 3.84	28.74 ± 3.13	< 0.001	25.41 ± 3.52	27.45 ± 3.40	< 0.001
WC (in cm)	95.40 ± 12.40	103.13 ± 9.40	< 0.001	89.20 ± 8.74	97.87 ± 6.59	< 0.001	83.75 ± 9.25	90.29 ± 8.34	< 0.001
SBP (mmHg)	122.40 ± 17.50	137.60 ± 17.60	< 0.001	129.92 ± 17.76	138.92 ± 16.16	< 0.001	126.88 ± 18.05	144.06 ± 16.52	< 0.001
DBP (mmHg)	75.90 ± 10.04	81.10 ± 11.50	< 0.001	80.28 ± 11.63	85.55 ± 10.19	< 0.001	78.19 ± 11.42	87.74 ± 10.91	< 0.001
FBG (mmol/l)	4.96 ± 0.75	5.75 ± 1.09	< 0.001	5.17 ± 0.84	5.72 ± 1.04	< 0.001	4.92 ± 0.52	5.21 ± 0.58	< 0.001
TC (mmol/l)	5.14 ± 1.06	5.28 ± 0.95	0.185	5.15 ± 0.98	5.25 ± 0.94	0.198	5.30 ± 0.98	5.63 ± 0.99	0.002
TG (mmol/l)	1.26 ± 0.57	1.78 ± 0.68	< 0.001	1.27 ± 0.58	1.71 ± 0.65	< 0.001	1.20 ± 0.62	1.83 ± 0.71	< 0.001
HDL (mmol/l)	1.18 ± 0.28	1.00 ± 0.26	< 0.001	1.13 ± 0.25	0.98 ± 0.21	< 0.001	1.34 ± 0.33	1.18 ± 0.28	< 0.001
LDL (mmol/l)	3.31 ± 0.94	3.44 ± 0.87	0.205	3.39 ± 0.90	3.44 ± 0.87	0.542	3.28 ± 0.82	3.59 ± 0.89	< 0.001
siMS score	2.62 ± 0.49	3.47 ± 0.54	< 0.001	2.67 ± 0.55	3.34 ± 0.50	< 0.001	2.49 ± 0.536	3.22 ± 0.58	< 0.001
Obesity Status	58.4%:41.6%	26.6%:73.4%	< 0.001	82.9%:17.1%	66.0%:34.0%	< 0.001	86.6%:13.4%	76.2%:23.8%	< 0.010
Diabetes Status	84.5%:15.5%	52.9%:47.1%	< 0.001	75.8%:24.2%	53.0%:47.0%	< 0.001	85.7%:14.3%	79.6%:20.4%	0.125
Hypertension Status	78.5%:21.5%	60.2%:39.8%	< 0.001	55.0%:45.0%	27.4%:72.6%	< 0.001	64.5%:35.5%	15.0%:85.0%	< 0.001

^a^ Ethnicity of the individuals forming each of the three study cohorts is presented. Each of the cohorts is sub-divided onto two subgroups: MetS, subgroup comprising individuals afflicted with metabolic syndrome; MetS-free, subgroup comprising individuals free of metabolic syndrome. N, the count of individuals forming each subgroup is indicated. The values for the quantitative phenotype traits are presented as mean ± standard deviation.

^b^ Unpaired Student’s *t*-test was used to compare means between the MetS-free and MetS groups. For these analyses, a *p-value* < 0.05 was considered significant.

The *CAV1* rs1997623 variant passed the quality control for HWE >10^–3^ in each of the three cohorts (Table 2). The minor allele frequency (MAF) varied across the three study cohorts (16,17% in Arabs and South Asians versus 10% in South East Asians). The sub-cohorts of MetS and MetS-free individuals from each of the three study cohorts had a varied MAF (21% versus 14% in sub-cohorts of Arab individuals). The 1000 Genomes Project Phase 3 allele frequencies for the minor allele A also showed significant variations across continental populations: Africans (31%), admixed Americans (11%), East Asians (7%), Europeans (14%), and South Asians (13%).

**TABLE 2:** Assessment of Hardy–Weinberg Equilibrium (HWE) deviation for rs1997623 genotypes across sample sets of MetS, MetS- free and All individuals among Arab, South Asian and South East Asian cohorts.

Population^a^	Group^b^	Minor allele	Major allele	Minor allele frequency	Genotype counts	O(HET) ^c^	E (HET) ^c^	HWE *p*-value^c^
Arab (N = 479)	ALL	A	C	0.166	14/131/334	0.273	0.276	0.742
	MetS	A	C	0.212	8/57/107	0.331	0.334	0.823
	MetS-free	A	C	0.140	6/74/227	0.241	0.241	1.000
South Asian (N = 660)	ALL	A	C	0.174	23/184/453	0.278	0.287	0.417
	MetS	A	C	0.190	9/63/141	0.295	0.308	0.511
	MetS-free	A	C	0.166	14/121/312	0.271	0.278	0.608
South East Asian (N = 362)	ALL	A	C	0.102	4/66/292	0.182	0.183	0.778
	MetS	A	C	0.114	1/31/113	0.214	0.207	0.695
	MetS-free	A	C	0.094	3/35/179	0.161	0.171	0.414

^a^ Ethnicity of the individuals forming each of the three study cohorts is presented along with the count of individuals forming the cohort.

^b^ ALL, all the individuals of the cohort were considered; MetS = only the individuals with metabolic syndrome were considered; MetS-free = only the individuals free of metabolic syndrome were considered.

^c^ O(HET) = observed frequency of heterozygous carriers; E (HET) = expected frequency of heterozygous carriers; Study variant passes the test for Hardy–Weinberg Equilibrium when the HWE *p*-value is > 10^–3^.

### *CAV1* rs1997623 C >A variant is significantly associated with siMS score and MetS status in arab adults

Linear regression tests adjusted for age and sex revealed significant associations from the study variant with three of the tested 12 quantitative metabolic traits *albeit* only in the Arab cohort (Table 3); such associations were with increased levels of siMS (effect = 0.206; *p*-value = 0.0035; P_*emp*_ = 0.0028); systolic blood pressure (effect = 4.029; *p*-value = 0.023; P_*emp*_ = 0.022) and diastolic blood pressure (effect = 2.669; *p*-value = 0.019; P_*emp*_ = 0.018). Of these, the *p*-value for the association with siMS score at 0.0035 passed the Bonferroni-corrected *p*-value threshold of 0.0042 (=0.05/12). Upon correcting the linear regression model for the confounding factor “drug treatment,” such as diabetes, hypertension, and lipids-lowering medications, the association with siMS score remained significant (effect = 0.18; *p*-value = 0.035; P_*emp*_ = 0.035).

**TABLE 3:** Summary statistics of quantitative trait association with rs1997623_A in Arab, South Asian and South East Asian cohorts using linear regression adjusted for Age and Sex.

Cohort	Arab			South asian			South east asian		
Trait	Effect Size	*p*-value	P_*emp*_ ^a^	Effect Size	*p*-value	P_*emp*_ ^a^	Effect Size	*p*-value	P_*emp*_ ^a^
siMS score^b^	**0.206**	**0.004**	**0.003**	-0.001	0.967	0.970	−0.014	0.869	0.869
SBP^b^	**4.029**	**0.023**	**0.022**	-0.754	0.523	0.527	1.633	0.451	0.456
DBP^b^	**2.669**	**0.019**	**0.018**	0.371	0.649	0.648	0.484	0.741	0.739
Height	−0.038	0.938	0.942	0.482	0.281	0.278	0.634	0.367	0.366
Weight	0.507	0.712	0.720	1.519	0.062	0.064	0.043	0.974	0.972
BMI	0.603	0.206	0.206	0.519	0.061	0.061	−0.211	0.631	0.630
WC	1.041	0.386	0.387	0.682	0.291	0.289	0.503	0.650	0.647
FBG	0.137	0.181	0.181	0.006	0.927	0.929	0.064	0.382	0.387
HDL	−0.046	0.099	0.098	0.022	0.189	0.190	0.001	0.975	0.975
LDL	0.033	0.730	0.729	0.048	0.456	0.466	−0.114	0.280	0.282
TC	−0.024	0.818	0.821	0.065	0.357	0.349	−0.195	0.104	0.100
TG	0.045	0.510	0.518	0.045	0.325	0.328	−0.110	0.207	0.208

^a^ Empirical *p-*values (P_*emp*_) were generated using the max(T) permutation procedure based on 10,000 permutations. A value of <0.05 is set for P_*emp*_ value to assess the statistical significance of the association signal.

^b^ Significant *p*-value and P_*emp*_ values are given in bold font.

Logistic regression analysis adjusted for age and sex revealed significant association with presence of MetS (Odds ratio = 1.811 [1.25–2.61]; *p*-value = 0.0015; P_*emp*_ = 0.0013) *albeit* only in the Arab cohort (Table 4). Associations with diabetes, obesity, or hypertension status were not seen at statistically significant levels in any of the three cohorts.

**TABLE 4:** Summary statistics of dichotomous traits association with rs1997623_A in Arab, South Asian and South East Asian cohorts using logistic regression adjusted for Age and Sex.

Cohort	Arab			South asian			South east asian		
Trait	OR [CI 95%]^a^	*p*-value	P_*emp*_ ^b^	OR [CI 95%]^a^	*p*-value	P_*emp*_ ^b^	OR [CI 95%]^a^	*p*-value	P_*emp*_ ^b^
MetS Status^c^	**1.811 [1.250–2.610]**	**0.002**	**0.001**	1.189 [0.880–1.600]	0.255	0.254	1.118 [0.673–1.850]	0.666	0.670
Diabetes Status	1.261 [0.836–1.910]	0.266	0.262	1.185 [0.850–1.640]	0.310	0.317	1.381 [0.720–2.630]	0.328	0.327
Obesity Status	1.247 [0.878–1.770]	0.217	0.223	1.129 [0.810–1.570]	0.473	0.480	0.600 [0.290–1.240]	0.170	0.170
Hypertension Status	1.286 [0.796–2.080]	0.304	0.313	0.920 [0.688–1.230]	0.584	0.587	1.176 [0.690–1.980]	0.542	0.546

^a^ Odds ratio (OR) and 95% confidence interval (CI) for risk of metabolic syndrome or diabetes or obesity or hypertension.

^b^ Empirical *p-*values (P_*emp*_) were generated using the max(T) permutation procedure based on 10,000 permutations. A value of <0.05 is set for P_*emp*_ value to assess the statistical significance of the association signal.

^c^ Significant *p*-value and P_*emp*_ values are given in bold font.

Results from power calculation are presented in Table 5. Results of power calculation indicated that the study cohorts had 80% power to detect associations with the CAV1 variant (MAF = 16.6% in Arab, 17.4% in South Asian, and 10.2% in South East Asian cohorts) and could explain 1.7% variance in the quantitative traits of siMS score, SBP and DBP of the Arab cohort (1.3% and 2.5% variance in the cases of South Asian and South East Asian cohorts, respectively). The observed effect sizes of 0.206, 4.03 and 2.67 in the Arab cohort for the association of the CAV1 variant with siMS score, SBP and DBP, respectively (see Table 3) were less than the expected effect sizes of 0.225, 4.69 and 2.73, respectively.

**TABLE 5:** Power Calculation for the Arab, South Asian and South East Asian cohorts for the traits of siMS score, SBP and DBP found associated in the Arab cohort.

Trait	R^2 marginal effect^a^	Sample size	Expected effect size^b^	Sample size	Expected effect size^b^	Sample size	Expected effect size^b^
		Arab cohort		South Asian cohort		South East Asian cohort	
siMS score	0.001	7,845	0.0547	7,845	0.0366	7,845	0.0492
	0.005	1,566	0.1223	1,566	0.0818	1,566	0.11
	0.009	868	0.1641	868	0.1097	868	0.1476
	***0.013***	600	0.1972	***600***	***0.1319***	600	0.1774
	***0.017***	***458***	**0.2255**	458	0.1508	458	0.2028
	0.021	370	0.2506	370	0.1676	370	0.2254
	***0.025***	310	0.2734	310	0.1828	***310***	***0.246***
	0.029	267	0.2945	267	0.1969	267	0.2649
	0.033	234	0.3142	234	0.2101	234	0.2826
	0.037	208	0.3327	208	0.2224	208	0.2992
	0.041	187	0.3502	187	0.2342	187	0.315
	0.045	170	0.3669	170	0.2453	170	0.33
	0.049	156	0.3828	156	0.256	156	0.3444
SBP	0.001	7,845	1.1388	7,845	1.2056	7,845	1.443
	0.005	1,566	2.5465	1,566	2.6957	1,566	3.2267
	0.009	868	3.4165	868	3.6167	868	4.329
	***0.013***	600	4.1061	***600***	***4.3468***	600	5.2028
	***0.017***	***458***	***4.6955***	458	4.9707	458	5.9497
	0.021	370	5.2188	370	5.5246	370	6.6127
	***0.025***	310	5.6941	310	6.0279	***310***	***7.215***
	0.029	267	6.1328	267	6.4922	267	7.7708
	0.033	234	6.5421	234	6.9255	234	8.2895
	0.037	208	6.9272	208	7.3332	208	8.7775
	0.041	187	7.292	187	7.7194	187	9.2398
	0.045	170	7.6395	170	8.0872	170	9.68
	0.049	156	7.9718	156	8.439	156	10.1011
DBP	0.001	7,845	0.6641	7,845	0.6748	7,845	0.9049
	0.005	1,566	1.4849	1,566	1.5088	1,566	2.0233
	0.009	868	1.9922	868	2.0243	868	2.7146
	***0.013***	600	2.3943	***600***	***2.4329***	600	3.2625
	***0.017***	***458***	***2.738***	458	2.7821	458	3.7308
	0.021	370	3.0431	370	3.0921	370	4.1466
	***0.025***	310	3.3203	310	3.3738	***310***	***4.5243***
	0.029	267	3.5761	267	3.6337	267	4.8728
	0.033	234	3.8148	234	3.8762	234	5.198
	0.037	208	4.0393	208	4.1044	208	5.5041
	0.041	187	4.2521	187	4.3205	187	5.7939
	0.045	170	4.4547	170	4.5264	170	6.07
	0.049	156	4.6485	156	4.7233	156	6.334

^a^ R^2 multiplied by 100 gives the percent phenotype variance in the quantative trait.

^b^ The values for R^2 and expected effect sizes for the given sample size of each of the three study cohorts are given in italics and bold font.

## Discussion

In the present study, we show that the *CAV1* rs1997623-A variant is significantly associated with metabolic syndrome, represented as a dichotomous trait (MetS) and as a quantitative trait of continuous metabolic syndrome score (siMS), in adult Arabs from Kuwait. The statistical significance of association was indicated by *p-values* passing the Bonferroni corrected thresholds and by significant empirical *p*-values. These observed associations complement the findings from our previous study on pediatric MetS in Arabs (Nizam et al., 2018).

We present for the first time the rs1997623 from *CAV1* as a risk variant for MetS. At the most, this variant is only in a weak LD with two other variants, namely rs11773845 and rs926198, associated with MetS by previously researchers as enumerated below: The *CAV1* rs11773845 was found to be consistently associated with high serum TG and MetS in Latin Americans (Mora-García et al., 2018), and *the CAV1* rs926198 was found to be correlated with MetS in Caucasians and Hispanics (Baudrand et al., 2015). Caucasians harboring the minor allele at rs926198 displayed higher odds of insulin resistance and low HDL. Our study variant *CAV1* rs1997623 is not in particularly strong LD with the above-discussed two variants rs11773845 and rs926198 (*R* ^2^ = 0.2281 and 0.2952, respectively). Thus, the presented study variant rs1997623 is a newly proposed risk variant of *CAV1* known for its association with MetS.

Genome annotation systems, such as Ensembl (Cunningham et al., 2022), annotate the studied variant to overlap as many as nine transcript isoforms of *CAV1 and* five protein isoforms of different lengths. Depending on the transcript and protein isoform, rs1997623 can be a missense (leading to amino acid change) or synonymous (leading to no change in amino acid) or an intronic or an upstream gene variation. The Ensembl genome annotation system annotates the variant as part of a regulatory region within the promoter.

It is intriguing that we do not find the association signal between the study variant and MetS in the South Asian and South East Asian individuals. The study variant, *CAV1* rs1997623, displays considerable variation (7%–31%) in allele frequency across the continents. The frequency of the rs1997623_A allele across the populations is 7% in East Asians, 11% in admixed Americans, 13% in South Asians, 14% in Europeans, and 31% in Africans. The frequency of the A-allele in our study cohort of Arab individuals is 16.6%, with the sub-cohort of individuals with MetS exhibiting a higher value of 21% compared to 14% exhibited by the sub-cohort of MetS-free individuals. Considerable regional and global variation in the estimates for MetS burden on both children (Noubiap et al., 2022) and adults (Ranasinghe et al., 2017; Moreira et al., 2020) have also been reported. These variations in the prevalence of MetS across the populations are probably consistent with variations in the effect allele frequencies and with the association signals across the populations. The prevalence rate of MetS is particularly high in Middle East countries and it is a noticeable cause for stroke, coronary heart disease, and cardiovascular disease in these countries (Ansarimoghaddam et al., 2018). Genetics of the Arab population in the Peninsula has been largely driven by consanguinity and inbreeding. A combination of inherent genetic predispositions and rapid lifestyle changes in the rich post-oil era has shaped the observed high prevalence of metabolic disorders in the region. Generalizing metabolic risk loci established in global populations to the Arab population and vice versa is an open topic and has been discussed, in our previous study (Hebbar et al., 2021), in the context of differences in allele frequencies, linkage disequilibrium, effect sizes, and heritability), and phenotype variance.

Interestingly, apart from targeted SNP association studies, there has been no large-scale GWA study performed implicating *CAV1* variants in the context of metabolic diseases in any ethnic group. Even the targeted SNP association studies, as mentioned earlier, examined only few SNPs from *CAV1* in the context metabolic traits and disorders. Therefore, our data, by way of showing that the association signal involving the studied variant is seen only in the Kuwaiti cohort and not in the cohorts of South Asians and South East Asians, demonstrates for the first time the ethnic-specific variations in *CAV1* impacting MetS.

As a limitation, the study cohorts are of moderate sample sizes at 479 for the Arab cohort, 660 for the South Asian cohort, and 362 for the South East Asian cohort. However, the reported associations in the Arab adults’ cohort are supported by significant empirical *p*-values and they replicate the findings from our studies on cohorts of Arab children. Considering that the study variant is a “common” variant with MAF values of 17% in Arab and South Asian cohorts and 10% in East Asian cohort, the above-mentioned sample sizes give sufficient power at 80% to the study to detect upto 1.7%, 1.3% and 2.5% phenotypic variance in siMS score of Arab, South Asian and South East Asian cohort, respectively. The study variant association in Arabs which is not observed in South Asian and South East Asian individuals living in Kuwait suggest a unique life style or environmental factor interaction with *CAV1* in Arabs mediating MetS. Nevertheless, it is fair to mention that future studies to replicate the reported ethnic-specificity may benefit from large multi-ethnicity cohorts with deep phenotyping relating to life style and environmental factors.

## Conclusion

The present study builds on our first discovery that demonstrated a significant association between rs1997623-A and pediatric MetS (Nizam et al., 2018). Here we replicated the same association in Kuwaiti adults. The study is the first to show the association of the *CAV1* rs1997623-A variant with MetS status and increased siMS score. Combined with our previous study, the association signals are seen in pediatric and adult MetS. We demonstrate the ethnic variation in the association signal—the association is seen only in the Kuwaiti Arab cohort and not in South Asian or South East Asian cohorts.

## Data Availability

The raw data supporting the conclusion of this article will be made available by the authors, without undue reservation.
